# Fuzzy multi-objective medical service organization selection model considering limited resources and stochastic demand in emergency management

**DOI:** 10.1371/journal.pone.0212308

**Published:** 2019-03-13

**Authors:** ShuJie Liao, Haiting Tu, Cheng Hu, Wulin Pan, Jianwu Xiong, Dongyang Yu, Lei Jing, Wei Pan

**Affiliations:** 1 Cancer Biology Research Center, Tongji Hospital, Tongji Medical College, Huazhong University of Science and Technology, Wuhan, Hubei, PR China; 2 School of Economic and Management, Wuhan University, Wuhan, China; 3 Management Science and Data Analytics Research Center, Wuhan University, Wuhan, China; Southwest University, CHINA

## Abstract

In recent years, most countries around the world have faced increasing pressures in the realm of emergency management than ever before. Medical service organization selection is one of the most vital facets of emergency management. Meanwhile, during the selection process, many criteria may conflict with one another and information is uncertain, rendering decision-making processes complex. Hence, multi-objective optimization, fuzzy way and stochastic theories serve as suitable means of addressing such problems. In this paper, a fuzzy multi-objective linear model is developed to overcome medical service organization selection issues and uncertain information. Meanwhile, a fuzzy objective and weight are applied to enable the decision-maker to select suitable schemes while considering stochastic medical service demand. Moreover, real data cannot been obtained. Hence, according to actual conditions, we assume relative information. For illustrative purposes, a numerical example is presented to verify the effectiveness of the proposed model from experimental data.

## 1. Introduction

In recent years, in the field of emergency management, many countries have been confronted with a lack of efficient emergency management and an increase in death tolls. Therefore, these countries must sustain heavy losses when disasters occur. For example, in 2008 the Chinese Wenchuan earthquake killed over 69,000 people, injured over 370,000 people, left over 17,900 people missing, causing economic losses of over 845.2 billion RMB; in 2010, the Haitian earthquake killed over 200,000 people, affecting over 1,000,000 people. From this perspective, emergency medical service organization selection has become extremely important to countries. In such environments, scholars and managers are more concerned about issues of emergency medical service organization selection than ever before.

In existing studies most scholars of emergency management have paid much more attention to issues of vehicle optimization, supply networks and so on. For example, Sheu proposed a means of designing a seamless centralized emergency supply network by integrating three subnetworks (the shelter, medical, and distribution networks) to support emergency logistics operations in response to large-scale natural disasters [1]. Wilson et al. described a novel combinatorial optimization model of this problem that acknowledges its temporal nature by employing a scheduling approach [2]. Cheng and Liang examined the locations of emergency rescue problem occurrence for urban ambulance and railway emergency systems [3]. Wohlgemuth et al. modeled a multistage mixed integer problem that is able to operate under variable demand and transport conditions [4].

Meanwhile, in practical situations, medical resource services are central to emergency management. Based on real conditions, Torres presented a novel multi-objective heuristic approach for the efficient distribution of 24-h emergency units [5]. Topaloglu constructed a multi-objective programming model for scheduling emergency medicine residents [6]. Walls established a multicenter registry and initiated the surveillance of a longitudinal, prospective convenience sample of intubations at 31 EDs [7]. This study is one of the first multicenter genetic research protocols designed solely for an Emergency Department (ED) [8]. Zakaria created a decision support system for the provision of emergency sanitation [9]. Cong studied family emergency preparedness plans for severe tornado events [10]. Canós improved emergency plan management systems using SAGA [11]. Zhu studied the standardized management of China's strategic railway emergency plan [12]. Calixto applied regional emergency plan requirements to the Brazilian case [13]. Su conducted a case study of emergency medical services deployment in Shanghai [14].

Moreover, as information is usually uncertain, researchers must consider stochastic data and the vagueness of input information. Araz established a fuzzy multi-objective covering-based vehicle location model for emergency services [15]. El-Ela established optimal preventive control actions using a multi-objective fuzzy linear programming technique [16]. Adan improved the operational effectiveness of tactical master plans for emergency and elective patients using stochastic demand and capacitated resources [17].

However, emergency medical service organization selection is a multicriteria decision-making problem affected by several conflicting factors including costs, degrees of social satisfaction, response times, service qualities, etc. The multiple criteria are usually unequally important. Moreover, information is usually uncertain. Consequently, scholars must analyze the trade-off among several criteria and the uncertainty of input information.

In real situations, objectives, constraints and weight information are usually uncertain. The decision-maker cannot precisely apply relative weights and information during emergency medical service organization selection. Meanwhile, stochastic emergency medical service demand also must be considered during medical service organization selection. However, most of the above models do not simultaneously consider such conditions. Thus, to generate a more practical and meaningful model for addressing the selection problem, we present a new fuzzy multi-objective medical service organization selection model based on stochastic demand and limited resources. In this model, objectives and weights are assumed to be fuzzy numbers with an interval fuzzy number where demand is stochastic.

This paper differs from past works in that it applies the following four conditions:

1. Due to vague information, we assume that objectives and some constraints are fuzzy.
2. Based on practical conditions, emergency medical demand is defined as a stochastic variable.
3. Delayed medical service cost are considered.
4. Medical organizations can provide multiple services.

As the main motivation of this study, as information is usually uncertain and as multiple medical services must be considered in real situations, we determine how to optimize emergency medical service organization selection in uncertain environments for the country. Furthermore, in this paper, a numerical example is used to illustrate the validation of the proposed method, as the explored problem is complex and difficult to address in real life. From this example, we demonstrate that the proposed method is valid.

The rest of this paper is organized as follows. In Section 2, a fuzzy multi-objective model and its formulation for the decision-making process are proposed in which the objectives are not equally important and have fuzzy weights. Subsequently, a general linear multi-objective programming model for this problem is formulated and some definitions and appropriate approaches to solving this decision-making problem are discussed. Section 3 presents the numerical example and describes the results. Finally, concluding remarks are given in Section 4.

## 2. Materials and Methods

### 2.1. Multi-objective medical service organization selection model with stochastic demand and limited emergency management resources

**Fig 1:**
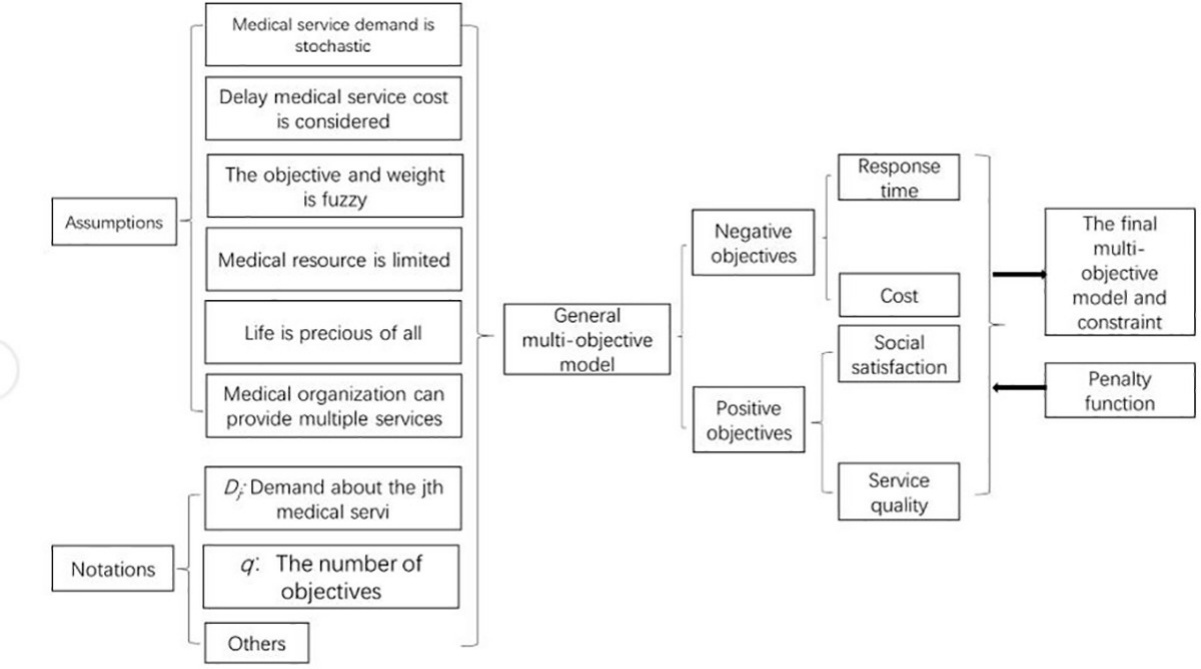
A flowchart of the model.

In emergency management, the manager receives medical resource demand information from the relevant department and allocates corresponding medical services for dealing with this risk. The challenge here is to allocate medial resource demand and select a suitable medical service organization approach. Notably, emergency medical service organization selection is a multiple criteria decision-making problem, and a multi-objective decision model must be built to allocate medical service demand for sudden risk and to select an organization approach among other potential approaches. Meanwhile, in developing similar models, researchers have rarely simultaneously considered stochastic demand, fuzzy objective and weight factors. Our model recognizes that these phenomena must be considered to address emergency plan selection problems. The following section discusses our model in detail and presents a flowchart describing the proposed model (Fig 1). We first make the following assumptions:

1. Medical service demand is stochastic.
2. Delayed medical service costs are considerable.
3. The objective and weight are fuzzy.
4. Medical resources are limited.
5. Life is the most precious resource of all.
6. Medical organization can support multiple services.

Moreover, we use the following notation throughout this paper.
*D*_*j*_: Demand for the jth medical service
*q*: The number of objectives

k: The number of negative objectives
*m*: The number of constraints
*n*: The number of emergency medical organizations
*m*_*t*_: Medical services of the tth medical organization, *t = 1*,*2*,*…*,*n*
*p*_*tj*_: The price of the tth medical organization’s jth service, *j = 1*,*2*,*…*,*m*_*t*_
*V*_*tj*_: The maximum capacity for the tth medical organization to execute the jth service
*R*_*tj*_: The probability of satisfaction with the j-th product offered by the t-th supplier
*x*_*tj*_: The purchase quantity of a tth medical organization jth service user, *x*_*tj*_*∈x*, *x = {x*_11_, *x*_12_,…, $x_{nm_{t}}$*}*
*y*_*tj*_: Decision variable determining if the tth medical organization delivering the jth service is selected or not where y_tj_ = 1 when the tth medical organization is selected to deliver the jth service and where y_tj_ = 0 otherwise
*c*_*tj*_: The unit delay cost for the jth service supplied by the tth medical organization
*E*(*x*_*tj*_): The penalty function for the jth service supplied by the tth medical organization
*f*: Objective function *f = {f*_*1*_,*f*_*2*_,*…*,*f*_*q*_*}*
Then, a new general multi-objective model can be written as follows:

minf(1);⋯;f(k) (1)

maxf(k+1);⋯f(q) (2)

with the following constraints:

$$x_{tj}\in g_{i}(x),\;g_{i}(x)=\left\{\mathrm{Pr}(\sum_{t=1}^{n}\sum_{j=1}^{m_{t}}a_{itj}x_{tj}\geq b_{i})\geq \beta_{i},\;i=1,\ldots ,m\right\}$$ (3)

where *f*(*1*), …, *f*(*k*) are the negative objectives or criteria-like cost, response time, etc. *f*(*k+1*), …, *f*(*q*) are positive objectives or criteria such as the degree of social satisfaction, service quality and so on; *X*_*tj*_, *t* = 1,…, *n j* = 1,…,*m*_*t*_ are nonnegative decision variables and *b*_*i*_, *i* = 1,…, *m* are independent continuous random variables with given distributions while *a*_*itj*_ represents the coefficient of the *t-th* decision variable in the *i-th* constraint, and *β*_*i*_ is the *i-th* pre-assigned probability level 0<*β*_*i*_≤1, i = 1, …, *m*.

Meanwhile, to solve an emergency medical organization selection problem, we assume that the objective includes the cost *f*_1_, degree of social satisfaction *f*_2_, response time *f*_3_ and service quality level *f*_4_ together with the major constraint that medical services can mostly satisfy stochastic demand. Each emergency medical organization has its own unit cost, social satisfaction history, response time record and service quality data.

However, we assume that life is the most precious resource of all and that costs must been considered. Moreover, medical services are limited. At the same time, in real situations, many factors that shape medical service demand cannot been fully addressed. According to such conditions, we must define a penalty function. Notably, delayed medical services result in losses. Diverse conditions can result in different costs. With a scenario analysis we construct two penalty functions as follows:

1. When a delayed medical service can be provided by another organization, the penalty function is equal to zero, i.e.,
  $$E(x_{tj})=0$$ (4)
2. When a delayed medical service can immediately be replenished, the penalty function is linear as follows:
  $$E(x_{tj})=c_{tj}x_{tj}$$ (5)

The objective function for costs can be written as follows:

$$f_{1}=\sum_{t=1}^{n}\sum_{j=1}^{m_{t}}p_{tj}y_{tj}x_{tj}+E(x_{tj})$$ (6)

which should minimize the cost of the product.

The objective function for the degree of social satisfaction is defined as:

$$f_{2}=\sum_{t=1}^{n}\sum_{j=1}^{m_{t}}R_{tj}y_{tj}x_{tj}$$ (7)

which should maximize the number of reliable units.

The aggregate performance measure for the response time objective function is defined as:

$$f_{3}=\sum_{t=1}^{n}\sum_{j=1}^{m_{t}}L_{tj}y_{tj}x_{tj}$$ (8)

which should minimize the response time.

The objective function for service quality is defined as:

$$f_{4}=\sum_{t=1}^{n}\sum_{j=1}^{m_{t}}F_{tj}y_{tj}x_{tj}$$ (9)

which should maximize service quality.

In turn, the final form of the multi-objective model for medical service organization selection is as follows:

$$f_{1}*=\min\sum_{t=1}^{n}\sum_{j=1}^{m_{t}}p_{tj}y_{tj}x_{tj}+E(x_{tj})$$ (10)

$$f_{2}*=\max\sum_{t=1}^{n}\sum_{j=1}^{m_{t}}R_{tj}y_{tj}x_{tj}$$ (11)

$$f_{3}*=\min\sum_{t=1}^{n}\sum_{j=1}^{m_{t}}L_{tj}y_{tj}x_{tj}$$ (12)

$$f_{4}*=\max\sum_{t=1}^{n}\sum_{j=1}^{m_{t}}F_{tj}y_{tj}x_{tj}$$ (13)

S.t.

$$P(\sum_{t=1}^{n}\sum_{j=1}^{m_{t}}d_{t}y_{tj}x_{tj}\geq D)\geq \beta$$ (14)

$$x_{tj}\leq C_{tj}$$ (15)

$$x_{tj}\geq 0\;\mathrm{and}\;\mathrm{integer}$$ (16)

Generally, managers do not have exact and complete information related to decision-making criteria and constraints that are fuzzy and stochastic in nature. A new fuzzy multi-objective medical service organization selection model is thus developed to address this problem.

In the new model, ~ denotes the fuzzy environment. ≳ used in the objectives and constraints denotes a fuzziness of ≥ i.e., approximately greater than or equal to, and ≲ linguistically denotes “essentially smaller than or equal to.”

### 2.2. A new fuzzy multi-objective medical organization selection model considering multiple services and stochastic demand for emergency management

Thus, by applying *c*_*it*_ and *σ*_*i*_ where *c*_*it*_>0 and 0<*σ*_*i*_<*β*_*i*_ as predetermined values set by the decision-maker, satisfaction constraints of the decision-maker can be stated as follows:

The decision-maker is fully satisfied if

$$\mathrm{Pr}(\sum_{t=1}^{n}a_{it}x_{t}\geq b_{i})\geq \beta_{i},i=1,\ldots ,m$$ (17)

The decision-maker is almost satisfied if

$$\sigma_{i}<\mathrm{Pr}(\sum_{t=1}^{n}a_{it}x_{t}\geq b_{i})<\beta_{i},\;\mathrm{Pr}(\sum_{t=1}^{n}\left(a_{it}+c_{it})x_{t}\right)\geq b_{i})>\beta_{i},\;i=1,\ldots ,\;m$$ (18)

The decision-maker is not satisfied if

$$pr(\sum_{t=1}^{n}a_{it}x_{t}\geq b_{i})\leq \sigma_{i}\;or\;pr(\sum_{t=1}^{n}\left(a_{it}+c_{it})x_{t})\geq b_{i}\right)\leq \beta_{i},i=1,\cdots ,m$$ (19)

then, the equivalent deterministic constraints for (17)–(19), respectively, are

$$\sum_{t=1}^{n}a_{it}x_{t}\geq F_{i}^{-1}(\beta_{i}),\;i=1,\ldots ,\;m,$$ (20)

$$\begin{array}{l} F_{i}^{-1}(\sigma_{i})<\sum_{t=1}^{n}a_{it}x_{t}<F_{i}^{-1}(\beta_{i}),\;i=1,\ldots ,m\;\mathrm{and}\\ F_{i}^{-1}(\beta_{i})<\sum_{t=1}^{n}(a_{it}+c_{it})x_{t},\;i=1,\ldots ,\;m, \end{array}$$ (21)

$$\begin{array}{l} \sum_{t=1}^{n}a_{it}x_{t}\leq F_{i}^{-1}(\sigma_{i}),i=1,\ldots ,\;m\;\mathrm{or}\\ \sum_{t=1}^{n}(a_{it}+c_{it})x_{t}\leq F_{i}^{-1}(\beta_{i}),i=1,\;\ldots ,\;m \end{array}$$ (22)

where $F_{i}^{-1}(•)$ is the inverse of the cumulative distribution function of random variable *b*_*i*_, *i* = 1,…,*m*.

Using the Bellman–Zadeh approach [18, 19], the fuzzy set objective functions *f*_*p*_ and constraints *g*_*i*_ are defined as

$$f_{p}=\left\{x,\mu_{f_{p}}(x)\right\},\;g_{i}=\left\{x,\mu_{g_{i}}(x)\right\},\;x\in L,\;p=1,\cdots ,q,\;i=1,\ldots ,\;m$$ (23)

where $\mu_{f_{p}}(x),\;\mu_{g_{i}}(x)|x\to \left[0,1\right]$ are the membership functions of objectives and constraints and where $\mu_{f_{p}}(x),\;\mu_{g_{i}}(x)$ are the degree to which x belongs to objectives and constraints. The fuzzy set objectives and constraints are thus uniquely determined by their membership function $\mu_{f_{p}}(x),\;\mu_{g_{i}}(x)$ and the range of the membership function is a subset of nonnegative real numbers whose value is finite and usually finds a place in interval [0,1].

From (23), it is possible to obtain the solution proving the maximum degree

$$\max\mu_{D}(x)=\underset{x\in L}{\max}\left\{\underset{p=1,\ldots ,q}{\min}\mu_{f_{p}}(x),\;\underset{i=1,\ldots ,m}{\min}\mu_{g_{i}}(x)\right\}$$ (24)

$$x^{0}=\arg\;\underset{x\in L}{\max}\left\{\underset{p=1,\ldots ,q}{\min}\mu_{f_{p}}(x),\;\mu_{g_{i}}(x)\right\}$$ (25)

**Fig 2:**
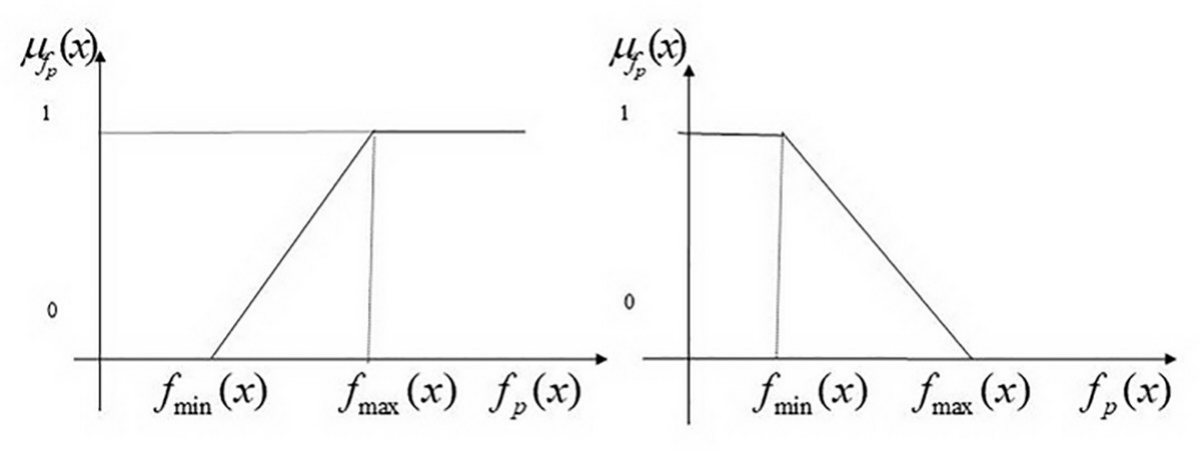
The different membership functions.

To obtain (24) and (25), it is necessary to build membership functions $\mu_{f_{p}}(x)\;\mathrm{and}\;\mu_{g_{i}}(x)$,

*p =* 1,…, *q*, *i* = 1,…, *m* from the corresponding *f*_*p*_(*x*), *g*_*i*_(*x*), *x*∈*L*, *p* = 1,…, *q*, *i* = 1, …, *m*. This function is satisfied through the use of membership functions

$$\mu_{f_{p}}(x)=\left\{ \begin{array}{l} 1,f_{p}(x)\geq \max_{x\in L}f_{p}(x)\\ \frac{f_{p}(x)-\min_{x\in L}f_{p}(x)}{\max_{x\in L}f_{p}(x)-\min_{x\in L}f_{p}(x)},\;\min_{x\in L}f_{p}(x)\leq f_{p}(x)\leq \max_{x\in L}f_{p}(x)\\ 0,f_{p}(x)\leq \min_{x\in L}f_{p}(x) \end{array}\right.$$ (26)

for maximized objective functions or through the use of membership functions as shown in Fig 2.

$$\mu_{f_{p}}(x)=\left\{ \begin{array}{l} 1,f_{p}(x)\leq \min_{x\in L}f_{p}(x)\\ \frac{\max_{x\in L}f_{p}(x)-f_{p}(x)}{\max_{x\in L}f_{p}(x)-\min_{x\in L}f_{p}(x)},\;\min_{x\in L}f_{p}(x)\leq f_{p}(x)\leq \max_{x\in L}f_{p}(x)\\ 0,f_{p}(x)\geq \max_{x\in L}f_{p}(x) \end{array}\right.$$ (27)

for minimized objective functions as shown in Fig 2.

The construction of (26) or (27) involves solving the following problems:

$$f_{p}(x)\to \min_{x\in L}f_{p}(x)$$ (28)

$$f_{p}(x)\to \max_{x\in L}f_{p}(x)$$ (29)

min_*x*∈*L*_*f*_*p*_(*x*), max_*x*∈*L*_*f*_*p*_(*x*) are obtained by solving the multi-objective problem as a single objective each time using only one objective where *x*∈*L* means that solutions must satisfy constraints. Since for every objective function *f*_*p*_(*x*) its value changes from min_*x*∈*L*_*f*_*p*_(*x*) to max_*x*∈*L*_*f*_*p*_(*x*), it may be considered a fuzzy number with the membership function $\mu_{f_{p}}(x)$ as shown in (26) and (27).

$$\mu_{g_{i}}(x)=\left\{ \begin{array}{l} 1,\;g_{i}(x)\geq F_{i}^{-1}(\beta_{i})\\ \min\left\{k_{i}(x),h_{i}(x)\right\},\;F_{i}^{-1}(\sigma_{i})<\sum_{t=1}^{n}a_{it}x_{t}<F_{i}^{-1}(\beta_{i})<\sum_{t=1}^{n}(a_{it}+c_{it})x_{t}\\ 0,\;g_{i}(x)\geq b_{i}+d_{i},\sum_{t=1}^{n}a_{it}x_{t}\leq F_{i}^{-1}(\sigma_{i})\;\mathrm{or}\;\sum_{t=1}^{n}(a_{it}+c_{it})x_{t}\leq F_{i}^{-1}(\beta_{i}) \end{array}\right.$$ (30)

where *k*_*i*_(*x*) and *h*_*i*_(*x*) are defined as

$$k_{i}(x)=(\sum_{t=1}^{n}a_{it}x_{t}-F_{i}^{-1}(\sigma_{i}))/(F_{i}^{-1}(\beta_{i})-F_{i}^{-1}(\sigma_{i})),\;i=1,\ldots ,\;m$$ (31)

$$h_{i}(x)=(\sum_{t=1}^{n}(a_{it}+c_{it})x_{t}-F_{i}^{-1}(\beta_{i}))/\sum_{t=1}^{n}c_{it}x_{t},\sum_{t=1}^{n}x_{t}\neq 0,\;i=1,\ldots ,\;m$$ (32)

$$\mathrm{On}\;\mathrm{the}\;\mathrm{other}\;\mathrm{hand},\;\mathrm{if}\;\mathrm{Pr}(\sum_{i=1}^{m}a_{it}x_{t}≲b_{i})≳\beta_{i},\;i=1,\;\ldots ,\;m$$ (33)

$$k_{i}(x)=(F_{i}^{-1}(1-\sigma_{i})-\sum_{t=1}^{n}a_{it}x_{t})/(F_{i}^{-1}(1-\sigma_{i})-F_{i}^{-1}(1-\beta_{i})),i=1,\ldots ,\;m$$ (34)

$$h_{i}(x)=(F_{i}^{-1}(1-\beta_{i})-\sum_{t=1}^{n}(a_{it}-c_{it})x_{t})/\sum_{t=1}^{n}c_{it}x_{t},\sum_{t=1}^{n}x_{t}\neq 0,\;i=1,\ldots ,\;m$$ (35)

(30) is a membership function of fuzzy values of linguistic variables that reflect constraints of a qualitative nature.

### 2.3. Decision-making processes

First, the max–min operator used by Zimmermann [20, 21] for fuzzy multi-objective problems is discussed. Then, the convex (weighted additive) operator that enables DMs to assign different weights to various criteria is described.

In fuzzy programming modeling, when using Zimmermann’s approach [22], a fuzzy solution is given by the intersection of all fuzzy sets representing either fuzzy objective or fuzzy constraints. The fuzzy solution for all fuzzy objectives and fuzzy constraints may be written as

$$\mu_{D}(x)=\left\{\left\{\overset{q}{\underset{p=1}{\cap }}\mu_{f_{p}}(x)\right\}\cap \left\{\overset{m}{\underset{i=1}{\cap }}\mu_{g_{i}}(x)\right\}\right\},x\in L$$ (36)

The optimal solution (x*) is given by

$$\mu_{D}(x^{*})=\max\mu_{D}(x)$$ (37)

where *μ*_*D*_(*x*), $\mu_{f_{p}}(x)$ and $\mu_{g_{i}}(x)$ represent membership functions of the solution, objective functions and constraints.

Under real conditions, different objectives and constraints are of unequal importance to DM and other patterns, and thus weight should be considered. The fuzzy weighted additive model can address this problem as described below.

The weighted additive model is widely used in vector-objective optimization problems; the basic premise is to use a single utility function to express the overall preference of DM to draw out the relative importance of criteria [23]. In this case, multiplying each membership function of fuzzy goals by its corresponding weight and then adding the results together generates a linear weighted utility function.

The fuzzy model proposed by Bellman and Zadeh and Sakawa and the weighted additive model developed by Tiwari are written as [24–26]

$$\mu_{D}(x)=\sum_{j=1}^{q}w_{j}\eta +\sum_{j=q+1}^{q+m}w_{j}\kappa$$ (38)

$$\sum_{p=1}^{q}w_{f_{p}}+\sum_{i=1}^{m}w_{g_{i}}=1,w_{f_{p}},w_{g_{i}}\geq 0$$ (39)

where $w_{f_{p}}$ and $w_{g_{i}}$ are the weighting coefficients denoting the relative importance of fuzzy goals and fuzzy constraints.

$$Max\;\tilde{w_{f_{p}}}\eta +\tilde{w_{g_{i}}}\kappa$$ (40)

$$\eta \leq \mu_{f_{p}}(x),\;p=1,\;\ldots ,\;q$$ (41)

$$\kappa \leq \mu_{g_{i}}(x),\;i=1,\;\ldots ,\;m$$ (42)

$$x_{t}\in L,\;t=1,\;\ldots ,\;n$$ (43)

If *w*_*j*_ denotes the fuzzy weight of the *j*-th objective or constraint, let $\tilde{w_{j}}=\{\underline{w_{j}},\;w_{j1},\;w_{j2},\;\bar{w_{j}}\}$, *j* = 1, *…*, *p+m* be a trapezoidal fuzzy number, or let $\tilde{w_{j}}=\{\underline{w_{j}},\;w_{j0},\;\bar{w_{j}}\}$ *j* = 1, …, *p+m* be a triangular fuzzy number. Then, by utilizing the *α*−*cut* approach for $\tilde{w_{j}}$, as a trapezoidal fuzzy number, the proposed model can be written in the form of a weighted max–min deterministic-crisp nonlinear programming model as follows:

$$\mathrm{Max}\sum_{j=1}^{q}w_{j}\eta_{j}+\sum_{j=q+1}^{q+m}w_{j}\kappa_{j}$$ (44)

s.t.:

$$\eta_{p}\leq \mu_{f_{p}}(x)p=1,\;\ldots ,\;q$$ (45)

$$\kappa_{i}\leq \mu_{g_{i}}(x)i=1,\;\ldots ,\;m$$ (46)

$$w_{j1}\leq w_{j}\leq w_{j2},j=1,..,m+p$$ (47)

$$\sum_{j=1}^{m+q}w_{j}=\sum_{p=1}^{q}w_{f_{p}}+\sum_{i=1}^{m}w_{g_{i}}=1,\;w_{f_{p}},\;w_{g_{i}}\geq 0$$ (48)

$$x_{t}\geq 0,\;t=1,\;\ldots ,\;n$$ (49)

$$\sum_{t=1}^{n}{E_{tj}x}_{tj}\leq E_{j}$$ (50)

$$\sum_{t=1}^{n}\sum_{j=1}^{m_{t}}{E_{tj}x}_{tj}\leq E$$ (51)

$$x_{tj}\leq C_{tj}$$ (52)

It should be noted that *w*_*j*_, *j = 1*,*2*,*…*,*m+p* become decision variables in addition to *η*_*p*_, *κ*_*i*_ and *x*_*t*_, *t =* 1, *…*, *n*. Constraint (48) ensures that the relative weights should add up to one. Additionally, fuzzy weights reflect the uncertain relative importance of objectives where the sum of all fuzzy weights should be one, i.e., $\sum_{j-1}^{m+q}w_{j}=1$, and where the sum of the lowest values of all fuzzy weights should be less than one, i.e., $\sum_{j-1}^{m+q}w_{j}\leq 1$. On the other hand, if for any *j-th* objective or constraint is considered a triangular fuzzy number, then *w*_*j*1_ and *w*_*j*2_ should be replaced with *w*_*j*0_ for the *j-th* objective or constraint.

## 3. Results and discussion

Since we cannot obtain real data on medical service organization selection, all data presented in this paper are experimental. In this section, for explaining validity of our model, we assume that all relative experimental data are based on real conditions. According to these experimental data, a numerical example is presented to illustrate the above listed model and algorithm. All experimental data used are shown in “Assumptions” and “Tables 1–7.”

### Assumptions

- Three medical services are purchased from three medical organizations. Factors such as price, satisfaction, response time, service quality and capacity can be obtained from historical data.
- Demands for three items are separately normally distributed fuzzy random variables with mean values of 100, 120 and 140, respectively, and variances of 25, 49 and 36, respectively; the three organizations’ services are limited.
- Objectives include the cost (*f*_1_), degree of social satisfaction (*f*_2_), response time (*f*_3_) and service quality (*f*_4_). All experimental data are included in Table 1 and Table 2. In Table 1, information on objectives and constraints is provided. As the only stochastic constraint, medical services must almost satisfy demand. Meanwhile, data on decision makers’ relative weights of fuzzy goals and constraints are shown in Table 2.

**Table 1:** Collected data for numerical example.

Medical organization	service kind	Price	Satisfaction	Response time	Service quality	Capacity
1	Item 1	3	0.8	10	0.7	80
	Item 2	7	08	11	0.8	100
	Item 3	4	0.9	12	0.9	120
2	Item 1	4	0.7	12	0.8	70
	Item 2	6	0.7	11	0.7	90
	Item 3	5	0.9	11	0.8	100
3	Item 1	3	0.9	11	0.9	120
	Item 2	6	0.7	10	0.6	110
	Item 3	5	0.9	12	0.8	130

**Table 2:** Weight values.

${\tilde{w}}_{1}$	0.2	0.25	0.3	0.5
${\tilde{w}}_{2}$	0.1	0.2	0.3	0.4
${\tilde{w}}_{3}$	0.1	0.2	0.4	0.5
${\tilde{w}}_{4}$	0.15	0.3	0.4	0.5
${\tilde{w}}_{5}$	0.25	0.3	0.5	0.6

From Eq (44)–(52) and relative experimental data we find that our numerical example has interesting implications for medical service organization schemes as illustrated in Table 3, which shows that the value of the penalty function does not always influence medical service organization selection. Regarding scenario descriptions, under one scenario, it is easy to find alternatives; under the other scenario, although a medical service can be purchased, there is a loss. Meanwhile, the loss is linearly related to the purchase volume. These scenarios serve as simplified descriptions of real situations. Thus, an interesting result occurs when a selected organization incurs a higher penalty than an unselected one. In Table 3 *x*_22_ and *x*_23_, we purchase more medical services at higher losses rather than incurring no loss. This phenomenon is usually inconsistent with what people anticipate. However, when costs are not the sole factor, we know that this is common. In our model, decisions are influenced by costs, degrees of social satisfaction and so on. This finding means that we must simultaneously consider multiple criteria requirements. A factor cannot always influence the final result. According to this rule, a penalty function cannot can occasionally impact medical service organization selection decisions due to other factors.

**Table 3:** Decision variable values about medical service under different penalty function.

Medical service Penalty function Penalty function	*E*(*x*_*tj*_) = 0	*E*(*x*_*tj*_) = *c*_*tj*_*x*_*tj*_
*x*_11_	0	0
*x*_21_	0	0
*x*_31_	105	105
*x*_12_	100	80
*x*_22_	27	47
*x*_32_	0	0
*x*_13_	120	120
*x*_23_	0	26
*x*_33_	26	0

Meanwhile, we use a sensitivity analysis to investigate the changes in optimal decision values regarding medical service Item 1 when only one parameter in the dataset changes while others remain unchanged. Relative data are shown in Tables 1 and 2. The computational results are illustrated in Tables 4–7.

**Table 4:** Sensitivity analysis for parameter price of medial organization 1 about item 1.

	Price of Medial organization 1 about Item 1	*x*_11_	*x*_21_	*x*_31_
1	3	0	0	105
2	4	0	0	105
3	5	0	0	105

**Table 5:** Sensitivity analysis for parameter satisfaction of medial organization 1 about item 1.

	Satisfaction of Medial organization 1 about Item 1	*x*_11_	*x*_21_	*x*_31_
1	0.7	0	0	105
2	0.8	0	0	105
3	0.9	0	0	105

**Table 6:** Sensitivity analysis for parameter response time of medial organization 1 about item 1.

	Response time of Medial organization 1 about Item 1	*x*_11_	*x*_21_	*x*_31_
1	10	0	0	105
2	11	0	0	105
3	12	0	0	105

**Table 7:** Sensitivity analysis for parameter service quality of medial organization 1 about item 1.

	Service quality of Medial organization 1 about Item 1	*x*_11_	*x*_21_	*x*_31_
1	0.7	0	0	105
2	0.8	0	0	105
3	0.9	0	0	105

From Tables 4–7 sensitivity analysis demonstrates that, when only one parameter is changed, the optimal decision is sometimes not influenced when applying current experimental data to the multi-objective problem. Moreover, this result simultaneously validates the result shown in Table 3 that the sole parameter occasionally fails to affect decisions.

The model proposed here is reasonable to apply under defined conditions and can solve the decision-making problem of selecting medical organization approaches with uncertain information. As the variables used in the model are different from real conditions, the model’s validity is limited to a certain extent. Therefore, it is necessary to adjust the variables used according to real conditions. In addition, this work mainly presents a theoretical analysis that needs to be verified with real data.

## 4. Conclusion

Medical service organization selection is one of the most important activities of emergency management. At the same time, medical service organization selection is a multiple objective decision-making problem for which objectives and constraints are not equally important. Moreover, information available to managers is uncertain. In considering these complex conditions and in solving this problem, we use fuzzy way, stochastic theory and multi-objective optimization to construct a medical service organization selection model. Hence, we develop a new fuzzy multi-objective model for this selection while considering stochastic demand. From the assumed data, we find that the proposed model can effectively address the uncertainties of input data and help managers identify suitable medical organization plans with a number of examples.

The examined problem can be transformed into a weighted max–min deterministic-crisp linear programming model. This transformation reduces the computational complexity and renders the application of fuzzy models more understandable. Finally, the proposed model’s further applications are also worthy of further research.

## References

[1] Sheu J.B; Pan C. A method for designing centralized emergency supply network to respond to large-scale natural disasters. *Transportation Research Part B*. 2014, 67, 284–305.

[2] Wilson D T.; Hawe G.I.; Coates Graham, Crouch R.S. A multi-objective combinatorial model of casualty processing in major incident response. *European Journal of Operational Research*. 2013, 230, 643–655.

[3] Cheng Y.H.; Liang Z.X. A strategic planning model for the railway system accident rescue problem. *Transportation Research Part E*. 2014, 69, 75–96.

[4] Wohlgemuth S.; Oloruntoba R.; Clausen U. Dynamic vehicle routing with anticipation in disaster relief. *Socio-Economic Planning Sciences*. 2012, 46, 261–271.

[5] Landa T.I.; Manjarres D.; Salcedo S.S.; Del S.J.; Lopez G.S. A multi-objective grouping Harmony Search algorithm for the optimal distribution of 24-hour medical emergency units. *Expert Systems with Applications*. 2013, 40, 2343–2349.

[6] Topaloglu S. A multi-objective programming model for scheduling emergency medicine residents. *Computers & Industrial Engineering*. 2006, 51, 375–388.

[7] Walls R.M.; Brown C.A.; Bair A.E.; Pallin D.J. EMERGENCY AIRWAY MANAGEMENT: A MULTI-CENTER REPORT OF 8937 EMERGENCY DEPARTMENT INTUBATIONS. *The Journal of Emergency Medicine*. 2011, 41, 347–354. 10.1016/j.jemermed.2010.02.024 20434289

[8] Lee D.C.; Peak D.A.; Jones J.S.; Domeier R.M.; Hendry P.L.; Rathlev N.K.; et al Variations in institutional review board reviews of a multi-center, Emergency Department (ED)–based genetic research protocol. *American Journal of Emergency Medicine*. 2013, 31, 967–969. 10.1016/j.ajem.2013.03.003 23623236 PMC3926443

[9] Zakaria F.; Garcia H.A.; Hooijmans C.M.; Brdjanovic D. Decision support system for the provision of emergency sanitation. *Science of the Total Environment*. 2015, 512–513, 645–658. 10.1016/j.scitotenv.2015.01.051 25662862

[10] Cong Z.; Liang D.A.; Luo J.J. Family Emergency Preparedness Plans in Severe Tornadoes. *Am J Prev Med*. 2014, 46, 89–93. 10.1016/j.amepre.2013.08.020 24355677

[11] Canós J.H.; Borges M.R.S.; Penadés M.C.; Gómez A.; Llavador M. Improving emergency plans management with SAGA. *Technological Forecasting & Social Change*. 2013, 80, 1868–1876.

[12] ZHU J.; WANG Z.Y.; QIN Y. Standardized Management of China Railway Emergency Plan Strategic Ponder. *Procedia Engineering*. 2013, 52, 701–706.

[13] Calixto E.; Larouvere E.L. The regional emergency plan requirement: Application of the best practices to the Brazilian case. *Safety Science*. 2010, 48, 991–999.

[14] Su Q.; Luo Q.Y.; Huang S.H. Cost-effective analyses for emergency medical services deployment: A case study in Shanghai. *Int*. *J*. *Production Economics*. 2015, 163, 112–123.

[15] Araz C.; Selim H.; Ozkarahan I. A fuzzy multi-objective covering-based vehicle location model for emergency services. *Computers & Operations Research*. 2007, 34, 705–726.

[16] El-Ela A.A.A.; Bishr M.; Allam S.; El-Sehiemy R. Optimal preventive control actions using multi-objective fuzzy linear programming technique. *Electric Power Systems Research*. 2005, 74, 147–155.

[17] Adan I.; Bekkers J.; Dellaert N.; Jeunet J.; Vissers J. Improving operational effectiveness of tactical master plans for emergency and elective patients under stochastic demand and capacitated resources. *European Journal of Operational Research*. 2011, 213, 290–308.

[18] Zadeh L.A. Fuzzy sets. *Information and Control*. 1965, 8, 338–353.

[19] Zadeh L.A. The concept of a linguistic variable and its application to approximate reasoning. *Information Sciences*. 1975, 8, 199–249.

[20] Zimmermann H.J. Fuzzy Set *Decision Making and Expert Systems*. 1987, Kluwer Academic Publishers, Boston.

[21] Zimmermann H.J. Fuzzy Set Theory and its Applications. 4th edition. 1993, Kluwer Academic Publishers, Boston.

[22] Zimmermann H.J. Fuzzy programming and linear programming with several objective functions. *Fuzzy Sets and Systems*. 1978,1,45–56.

[23] Lai Y.J.; Liu T.Y.; Hwang C.L. TOPSIS for MODM. *European Journal of Operational Research*. 1994, 76, 486–500.

[24] Bellman R.E.; Zadeh L.A. Decision-Making in a Fuzzy Environment. *Journal of Management Science*. 1970, 14(4):141–161.

[25] Sakawa M. Fuzzy Sets and Interactive Multiobjective Optimization. Plenum Press 1993, New York.

[26] Tiwari R.N.; Dharmar S.; Rao J.R. Fuzzy goal programming—An additive model. *Fuzzy Sets and Systems*. 1987, 24, 27–34.

